# Body Composition Characteristics of Community-Dwelling Older Adults With Dynapenia

**DOI:** 10.3389/fnut.2022.827114

**Published:** 2022-04-25

**Authors:** Hungu Jung, Shigeharu Tanaka, Ryo Tanaka

**Affiliations:** ^1^Graduate School of Humanities and Social Sciences, Hiroshima University, Hiroshima, Japan; ^2^Department of Sports, Health and Well-Being, Faculty of Human Health Science, Hiroshima Bunka Gakuen University, Hiroshima, Japan; ^3^Physical Therapy Major, School of Rehabilitation, Kanagawa University of Human Services, Yokosuka, Japan

**Keywords:** bioelectrical impedance analysis (BIA), body fat, leg, muscle function, muscle mass

## Abstract

This study aimed to determine the differences in muscle and fat masses of the arm and leg between older adults with dynapenia, sarcopenia, or presarcopenia and normal individuals. The percent body fat, lean body mass, and skeletal mass index were measured with bioelectrical impedance analysis. Muscle function was evaluated using grip strength and walking speed. Participants were classified into four categories. Dynapenia was defined as low muscle function with normal muscle mass. Sarcopenia was defined as the presence of both low muscle mass and low muscle function. Presarcopenia was defined as low muscle mass with normal muscle function. Control was defined as normal muscle mass and function. Multivariate analyses of variance were performed separately for women and men to test the main effect of sarcopenia category on body composition. Among the 356 enrolled participants, 270 were women, and 86 were men. In older women, the dynapenia and sarcopenia groups had significantly less muscle mass in the leg than the control group. In older men, the dynapenia group demonstrated a higher body fat mass in the leg than the control group. These results suggest that different strategies are necessary to prevent dynapenia in women and men.

## Introduction

Dynapenia is defined as an age-related loss in muscle strength in older adults that negatively affects their medical conditions, including metabolic syndrome (1), dementia (2), or mortality (3). Additionally, dynapenia is associated with abdominal obesity that worsens older adults' ability to perform instrumental activities of daily living over time (4). Although dynapenia is prevalent in older adults with reduced skeletal muscle mass, a specific population exhibits the loss of muscle strength despite having normal skeletal muscle mass (5). Based on the classification of sarcopenia suggested by the European Working Group on Sarcopenia in Older People (6), recent studies have defined dynapenia as the loss of muscle strength with normal muscle mass (7–11) to differentiate this condition from sarcopenia when describing the age-related loss of muscle mass and function. In a previous study, dynapenia prevalence rate was 10% among 213 Japanese older adults aged ≥60 years (12), whereas sarcopenia prevalence rate was 5.5–25.7% in previous studies involving Japanese older adults (13).

Dynapenia, which is mediated by physiological neuromuscular adaptations, is influenced by increases in body fat with consequent infiltration of intramuscular fat; thus, the loss of muscle function is not merely a result of sarcopenia (14–18). A previous study evaluating skeletal muscle characteristics in dynapenia, sarcopenia, and presarcopenia demonstrated that skeletal muscle characteristics of dynapenia vary markedly from those of presarcopenia, which is defined as having low skeletal muscle index but normal muscle function (11). The abovementioned study also indicated that older adults diagnosed with dynapenia or sarcopenia have a lower knee extension torque than both normal older adults and those with presarcopenia (but not those with both dynapenia and sarcopenia) (11). Additionally, a cross-sectional study showed that older adults with dynapenia had decreased thicknesses of the rectus femoris and medial gastrocnemius muscles compared with those without dynapenia (7). Another study reported that older adults with dynapenia have a higher body mass index and fat mass than adults with sarcopenia and presarcopenia (8). The extremities may also have varying muscle and fat masses in dynapenia, sarcopenia, and presarcopenia compared with normal older adults. Moreover, it may be different in men and women, as men have a higher ratio of muscle to fat than women (19). However, appendicular muscle and fat masses of older adults with dynapenia, sarcopenia, or presarcopenia have not been compared with those of normal older adults.

Therefore, this study aimed to evaluate the difference in appendicular muscle and fat masses of the arm and leg between older adults with dynapenia, sarcopenia, or presarcopenia and normal older adults. We hypothesized that skeletal muscle and fat masses of limbs would vary significantly between older adults with dynapenia, sarcopenia, or presarcopenia and those without dynapenia or sarcopenia.

## Methods

### Study Design

The study design was cross-sectional. Data on socio-demographic information (age, gender), body composition, and physical function were collected. All parameters were essential for assessing the participants' functional status and were not harmful. All procedures were conducted in accordance with the World Medical Association Declaration of Helsinki Ethical Principles for Medical Research Involving Human Subjects of 1975. This research has been approved by the institutional review board of the author's affiliated institution (IRB number 02-05).

### Setting

The participants were recruited through the staff of community centers, regional comprehensive support centers, and gymnasiums in the Hiroshima Prefecture in Japan. A flyer with an outline of the survey was also used for recruitment. Recruitment and data collection were performed between November 2020 and December 2020.

### Participants

Participants included in the study were (1) community-dwelling persons aged 65 years or older, and (2) persons with independent mobility [Barthel Index mobility score > 10 (full score 20)] (20). The exclusion criteria were as follows: (1) suspected cognitive impairment [mini-mental state test <23 (full score 30)] (21) and (2) serious illness (unstable cardiovascular disease, stroke, severe respiratory impairment, Parkinson's disease, diabetic peripheral neuropathy, or rheumatoid/arthritis). All participants provided written informed consent. People with suspected cognitive impairment were excluded to ensure the accuracy of the responses to the questionnaire survey. People with suspected serious diseases were excluded to prevent falls during measurement and to avoid worsening of diseases.

### Body Composition

Lean body mass (LBM) and body fat mass (BFM) were measured using the bioelectrical impedance analysis (BIA) method. The BIA was performed for both arms and legs. InBody 270 (InBody Japan Inc., Tokyo, Japan) was used to measure the LBM, BFM, and appendicular skeletal mass index (ASMI)—calculated as the skeletal muscle mass of the extremities—and then normalized by height squared (m^2^) (22). InBody 270 is an acceptable device for body composition analysis (23). Participants' hands and feet were wiped down with InBody tissues to increase their conductivity. Participants' feet were aligned with the foot electrodes. Their thumbs were placed on the oval electrodes. Participants needed to keep their arms straight and hold the handles away from their bodies at a 45° angle. They had to stay still and maintain the testing posture until the test was completed. The weight measured was subtracted by 0.8 kg, considering the garment weight. The examiner in charge of measuring body composition received training to use InBody 270, and thus, the examiner was familiar with the technique. Measurements were not necessarily performed in the fasting state.

In this study, the LBM and BFM represented the indices of muscle mass. Each site-specific LBM (kg) was divided by the height squared (m^2^) and used as the value for each region (kg/m^2^) in subsequent statistical analyses. The mean values of the left and right sides were used for analysis.

### Sarcopenia Category

**Figure 1:**
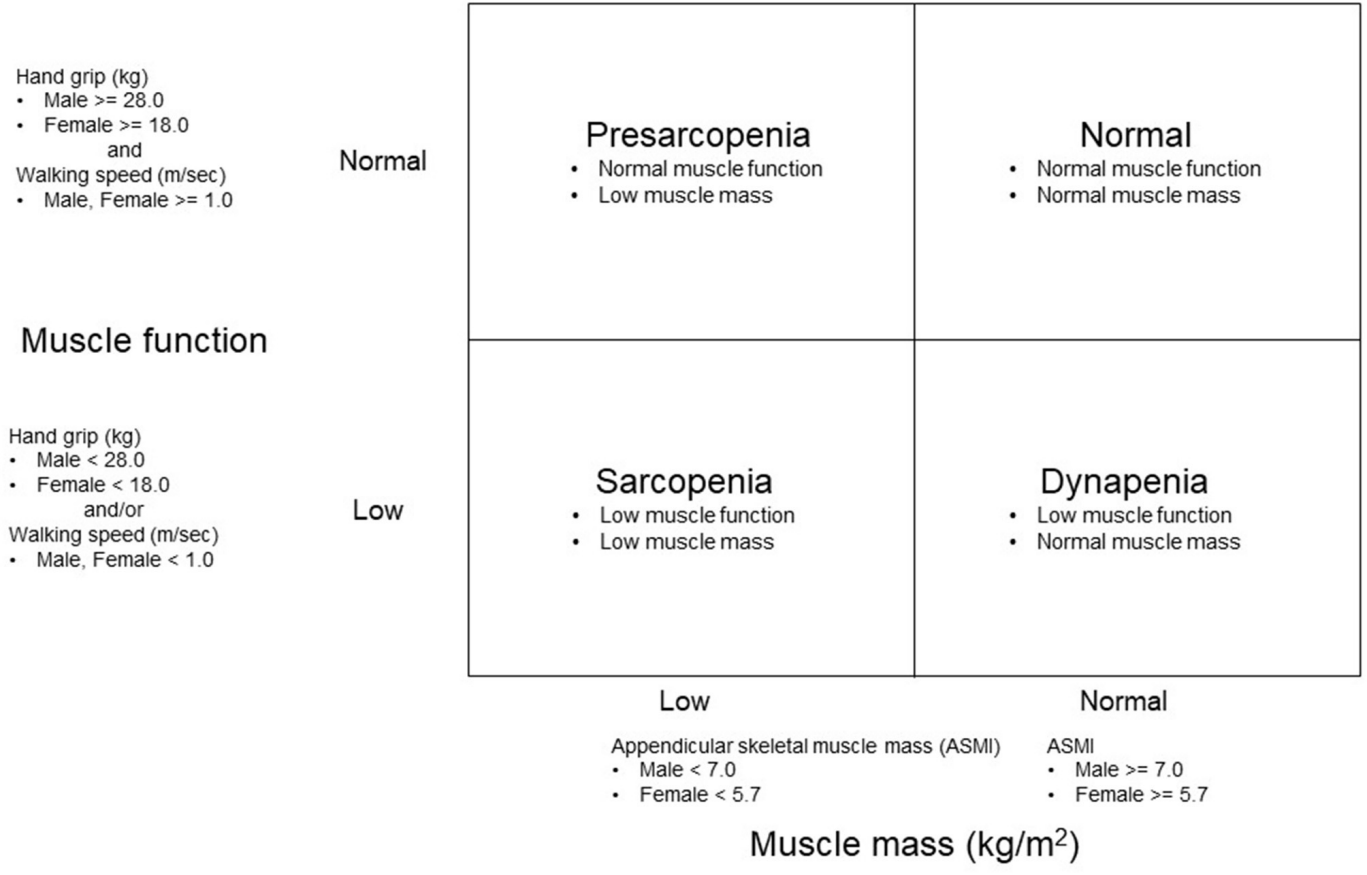
Methods of grouping based on an appendicular skeletal mass index (ASMI) and muscle function.

Muscle mass and function were used as indicators to classify the participants into four groups (Figure 1). ASMI represented the index of muscle mass. Grip strength (muscle strength) and walking speed (physical performance) were used to determine the presence of low muscle function. Sarcopenia categories were classified into four groups: dynapenia, sarcopenia, presarcopenia, and control. We defined dynapenia as low muscle function with normal muscle mass (11). Low muscle function was defined as having weak handgrip strength and/or slow walking speed (13). Sarcopenia was operationally defined using the diagnostic algorithm of the Asian Working Group for Sarcopenia (AWGS) based on the presence of both low muscle mass and function (13). Presarcopenia was defined as low muscle mass with normal muscle function (14). Control was defined as having normal muscle mass and function.

For the measurement of walking speed as a physical performance, the test participant walked along a 5-meter walking path according to the instructions (24). A spare path of 1 m was provided in front of the walking path. The teaching was unified with "Walk forward as if you were always walking.” Measurements were taken to the first decimal place and rounded to the second decimal place. Walking speed was measured only once.

For the measurement of grip strength as an indicator of muscle strength, we used a grip strength meter (TKK 5401 Grip-D; Takei, Niigata, Japan) (25). The test participant grasped the grip strength meter so that the pointer of the meter was on the outside. Before the measurement, the interphalangeal joints of the fingers were adjusted so that they were almost at right angles.

Measurements were taken once on both sides. Records were made in kilograms, and those less than a kilogram were rounded down. The record of the better left or right side was used for subsequent analysis.

### Statistical Analysis and Sample Size

The multivariate analysis of variance (MANOVA) was performed to evaluate the main effect of sarcopenia category on body composition (LBM of arm, LBM of leg, BFM of arm, and BFM of leg). We did not use the analysis of variance (ANOVA) test because multiple ANOVAs increase type 1 error. MANOVAs were performed separately for women and men. The main effects of the sarcopenia category on LBM and BFM, respectively, were examined after confirming a significant main effect of the sarcopenia category in the MANOVA. For body composition, for which the main effect was significant, multiple comparisons were performed using the Dunnett's method to compare the values of the control and dynapenia, sarcopenia, or presarcopenia groups. The significance level was set at 5%. JMP^®^ Pro ver. 16.0.0 (SAS Institute Inc., Cary NC, USA) was used for all statistical analyses.

The sample size required to ensure sufficient power in the MANOVA was calculated. G^*^power 3.1.9.7 was used for this calculation (26). The parameters were set as follows: effect size = 0.15, significance level = 0.05, effect size = 0.80, number of groups = 4, and response variables = 4. The result of the calculation demonstrated that a sample of 44 people (11 people in each group) was required to correctly detect a significant difference among the groups.

## Results

The study included 356 participants (270 women and 86 men). The socio-demographic information of the participants is shown in Table 1. No missing data were handled. The numbers and percentages of people in each sarcopenia category were as follows: 65 participants in the sarcopenia group (47 women, 17.4%, 18 men, 20.9%), 96 participants in the presarcopenia group (78 women 28.9%, 18 men 20.9%), 52 participants in the dynapenia group (36 women 13.3%, 16 men 18.6%), and 143 participants in the control group (109 women 40.4%, 34 men 39.6%).

**Table 1:** Background information.

**(A) Women**						
		**Control**	**Dynapenia**	**Sarcopenia**	**Presarcopenia**	***F*-value**
Number		109	36	47	78	
		40.4%	13.3%	17.4%	28.9%	
Age	(ys)	72.0	77.0^**^	77.9^**^	74.0^*^	16.753
	4.4	6.8	6.3	5.3	
Walking speed		1.34	1.00^**^	1.01^**^	1.36^**^	44.915
	(m/sec)	0.21	0.28	0.28	0.20	
Grip strength		23.4	17.6^**^	17.3^**^	21.4^**^	58.729
	(kg)	3.3	4.7	2.8	2.0	
BMI	(kg/m^2^)	23.8	24.8	21.0^**^	21.0^**^	27.172
		2.9	3.8	2.7	2.4	
LBM	(kg)	35.8	34.9	30.5^*^	31.1^*^	83.249
(whole body)		3.0	2.6	1.9	1.7	
LBM	(kg)	1.7	1.7	1.4^*^	1.4^*^	61.535
(right arm)		0.3	0.2	0.2	0.2	
LBM	(kg)	1.7	1.7	1.4^*^	1.4^*^	54.649
(left arm)		0.3	0.2	0.2	0.2	
LBM	(kg)	16.2	15.9	13.7^*^	14.1^*^	58.535
(trunk)		1.6	1.4	1.1	1.0	
LBM	(kg)	5.7	5.2^*^	4.4^*^	4.8^*^	83.261
(right leg)		0.6	0.6	0.5	0.5	
LBM	(kg)	5.7	5.2^*^	4.4^*^	4.7^*^	81.451
(left leg)		0.6	0.7	0.5	0.4	
ASMI	(kg/m^2^)	6.17	6.07	5.18^**^	5.30^**^	135.017
		0.43	0.37	0.34	0.25	
BFM	(kg)	19.2	19.6	14.3^*^	15.4^*^	14.079
(whole body)		5.6	6.9	5.6	4.5	
BFM	(kg)	1.3	1.4	1.0^*^	1.1^*^	9.913
(right arm)		0.5	0.7	0.5	0.3	
BFM	(kg)	1.4	1.4	1.0^*^	1.1^*^	10.106
(left arm)		0.5	0.7	0.5	0.4	
BFM	(kg)	9.2	9.6	6.7^*^	7.3^*^	13.768
(trunk)		3.0	3.5	3.1	2.5	
BFM	(kg)	3.1	3.1	2.3^*^	2.5^*^	15.315
(right leg)		0.8	1.0	0.8	0.7	
BFM	(kg)	3.1	3.0	2.3^*^	2.5^*^	15.111
(left leg)		0.8	1.0	0.8	0.7	
Total body	(kg)	27.9	27.3	23.9^*^	24.3^*^	83.072
water		2.3	2.0	1.5	1.3	
**(B) Men**						
		**Control**	**Dynapenia**	**Sarcopenia**	**Presarcopenia**	***F*** **-value**
Number		34	16	18	18	
		39.6%	18.6%	20.9%	20.9%	
Age	(ys)	73.8	75.6	81.9^**^	78.3^*^	9.664
	4.1	5.7	5.5	6.9	
Walking speed		1.28	0.88^*^	1.08^*^	1.19^*^	16.602
	(m/sec)	0.16	0.14	0.29	0.18	
Grip strength		36.1	35.0	24.7^**^	31.6^**^	21.513
	(kg)	4.1	7.4	5.6	3.2	
BMI	(kg/m^2^)	23.7	25.3	22.1^*^	21.2^**^	11.635
		2.4	2.2	2.1	2.1	
LBM	(kg)	47.3	45.1	37.6^*^	40.7^*^	31.258
(whole body)		4.2	3.7	3.8	2.6	
LBM	(kg)	2.7	2.6	1.9^*^	2.2^*^	29.435
(right arm)		0.4	0.4	0.3	0.2	
LBM	(kg)	2.6	2.6	1.8^*^	2.1^*^	36.036
(left arm)		0.4	0.3	0.3	0.2	
LBM	(kg)	22.1	21.3	16.7^*^	18.9^*^	33.610
(trunk)		2.2	2.1	2.0	1.2	
LBM	(kg)	7.8	7.3	6.1^*^	6.7^*^	18.764
(right leg)		0.8	0.7	1.0	0.6	
LBM	(kg)	7.8	7.2	6.0^*^	6.6^*^	20.708
(left leg)		0.8	0.7	1.0	0.6	
ASMI	(kg/m^2^)	7.47	7.46	6.29^**^	6.48^**^	38.998
		0.41	0.47	0.66	0.27	
BFM	(kg)	16.3	18.9	15.5	14.1	2.542
(whole body)		5.4	4.6	5.1	4.8	
BFM	(kg)	1.0	1.2	1.0	0.9	1.841
(right arm)		0.4	0.4	0.4	0.4	
BFM	(kg)	1.0	1.2	1.1	0.9	1.590
(left arm)		0.4	0.4	0.4	0.4	
BFM	(kg)	8.3	9.7	7.4	6.9	2.948
(trunk)		3.2	2.5	2.7	2.8	
BFM	(kg)	2.5	2.8	2.5	2.2	2.274
(right leg)		0.7	0.7	0.8	0.6	
BFM	(kg)	2.4	2.8	2.5	2.2	2.265
(left leg)		0.7	0.7	0.8	0.6	
Total body	(kg)	36.9	35.2	29.4^*^	31.8^*^	30.656
water		3.3	2.8	2.9	2.1	

* *p < 0.05;*

** *p < 0.01 (Multiple comparisons were performed using the Dunnett's method to compare the values of the control and dynapenia, sarcopenia, or presarcopenia groups).*

*BMI, body mass index, ASMI, appendicular skeletal mass index; LBM, lean body mass; BFM. body fat mass.*

*The values in the bottom row represent standard deviations*.

The body composition for each sarcopenia category is shown in Table 2. In women, the results of the MANOVA showed that the main effect of sarcopenia category on body composition was significant [*F*_(12)_ = 28.173, *p* < 0.01]. The dynapenia and sarcopenia groups had significantly less muscle mass in the leg than the control group. The sarcopenia group had significantly less muscle mass in the arm than the control group, whereas dynapenia group had significantly more muscle mass in the arm than the control group. Additionally, the sarcopenia group had significantly less BFM both in the arm and leg than the control group.

**Table 2:** Body composition for each sarcopenia category.

**(A) Women**						
		**Control**	**Dynapenia**	**Sarcopenia**	**Presarcopenia**	***F*-value**
LBM	(kg/m^2^)					
	Arm	0.72	0.76^*^	0.61^**^	0.60^**^	45.306
		0.10	0.09	0.09	0.07	
	Leg	2.37	2.27^**^	1.98^**^	2.05^**^	127.115
		0.15	0.16	0.13	0.10	
BFM	(kg/m^2^)					
	Arm	0.57	0.63	0.46^**^	0.47^**^	7.726
		0.21	0.31	0.20	0.16	
	Leg	1.29	1.34	1.03^**^	1.10^**^	10.129
		0.34	0.48	0.36	0.31	
**(B) Men**						
		**Control**	**Dynapenia**	**Sarcopenia**	**Presarcopenia**	***F*** **-value**
LBM	(kg/m^2^)					
	Arm	0.96	0.98	0.73^**^	0.79^**^	26.980
		0.11	0.12	0.12	0.07	
	Leg	2.78	2.75	2.41^**^	2.45^**^	24.523
		0.14	0.17	0.28	0.10	
BFM	(kg/m^2^)				
	Arm	0.36	0.46	0.42	0.33	2.949
		0.15	0.16	0.14	0.12	
	Leg	0.88	1.08^**^	1.00	0.82	3.945
		0.24	0.27	0.30	0.22	

* *p < 0.05;*

** *p < 0.01 (Multiple comparisons were performed using the Dunnett's method to compare the values of the control and dynapenia, sarcopenia, or presarcopenia groups).*

*LBM, lean body mass; BFM, body fat mass.*

*The values in the bottom row represent standard deviations*.

In men, the results of the MANOVA also showed that the main effect of sarcopenia category on body composition was significant [*F*_(12)_ = 9.073, *p* < 0.01]. The muscle mass of the sarcopenia group both in the arm and leg was less than that of the control group. However, unlike in women, no significant differences in the arm and leg muscle mass were detected between the control and dynapenia groups. Instead, BFM was higher in the leg in the dynapenia group compared to the control group.

## Discussion

This study focused on the differences in appendicular muscle and fat masses of the arm and leg between older adults with dynapenia, sarcopenia, or presarcopenia and normal older adults. Our results revealed significant differences in muscle mass of the leg between the control and dynapenia or sarcopenia groups in older women and in the BFM of the leg between the control and dynapenia groups in older men.

To the best of our knowledge, this study is the first to evaluate differences in muscle and fat masses for limbs among older women and men between normal individuals and those with dynapenia. A recent study involving 765 community-dwelling older adults reported that individuals with dynapenia tend to have the highest BFM among the four groups, but the sexes were not analyzed separately (8). Another study showed that older adults with dynapenia had decreased thicknesses of the rectus femoris and medial gastrocnemius muscles compared with individuals without dynapenia (7). Similarly, older adults with dynapenia show significantly decreased knee extension strength than normal older adults (11). Our data support the abovementioned findings and provide new evidence. Muscle mass in the leg measured using BIA was significantly decreased in the dynapenia group than in the control group, despite having described dynapenia with normal muscle mass, particularly in older women. This new finding suggests that low muscle mass in the leg might be overlooked in older women who are judged not to have sarcopenia on the basis of the AWGS criteria using the ASMI.

Muscle mass of the leg in older women was significantly decreased in the dynapenia group compared with that in the control group. Women have a higher prevalence of low bone mass at either skeletal site compared with bone mass in men (27). Moreover, the older the woman, the higher the prevalence of bone mass loss (27). Older women with very low bone mass demonstrated a significantly lower leg SMI than individuals with a lower bone mass; in other words, older women with low bone mass have low muscle mass (28). Our results indicate that decreased muscle mass of the lower limbs in older women should be carefully considered in dynapenia, even if it is defined as low muscle function with normal muscle mass based on ASMI.

In older men, BFM was higher in the leg in the dynapenia group than in the control group. Particularly in the leg, this finding is similar to that of a previous study showing that older adults with dynapenia have higher echo intensities in the quadriceps femoris muscles than normal older adults, demonstrating higher fat mass (11). An increase in fat infiltration within skeletal muscle in the leg leads to low muscle strength and physical performance (29–31). Our results suggest that increases in the fat mass of the leg should be viewed with caution in older men.

Our results have important implications for dynapenia. A study evaluating the quantity/quality of leg muscles reported that thigh muscle volume and fat mass were better indicators of SMI and low muscle function, respectively (11). Furthermore, calf circumference evaluation is used to diagnose sarcopenia or SMI (32). From a clinical perspective, the present study specifically suggests strategies for preventing dynapenia separately for females and males, including increasing muscle mass and decreasing fat mass in the leg of older women and men, respectively.

This study had several limitations. First, the present study had a cross-sectional design. Further longitudinal studies are needed to ascertain whether reductions in muscle mass and increases in fat mass of the leg influence the prevalence of dynapenia, sarcopenia, and presarcopenia over time. Second, we measured walking speed only once to minimize testing stress on participants. The AWGS recommends calculating the walking speed on an average of two tests (13). Additionally, walking speed measurements showed excellent test–retest reliability (24). Therefore, the effect of the walking speed values on the results of this study might be small. Third, we have no data of older adults regarding their physical activities or the performance of other physical tests such as the 5-time chair stand test. Therefore, we could not show that the differences in body composition between the control and dynapenia groups might be because of the differences in physical activity or test performance. Fourth, this study did not examine the muscle quality of older adults with dynapenia, sarcopenia, and presarcopenia. However, we showed the difference in the body composition of the arm and leg between normal older adults and those with dynapenia. Fifth, this study did not use dual-energy X-ray absorptiometry (DXA), the gold-standard technique in analyzing body composition. BIA methods used in this study strongly correlate with DXA methods, as reported in a previous study (33). Thus, the results were considered reliable. Additionally, attention should be paid to the data obtained using different devices to measure body composition as there is a slight difference in the values acquired through different measuring devices. Finally, we did not always measure body composition in the fasting state. Although body fluid volume variance by measuring time affects the body composition of the trunk, that of the arm and leg was not affected (33). Therefore, the results for ASMI might not have been affected.

## Conclusions

Older women with dynapenia had less muscle mass in the leg than normal older women. In contrast, older men with dynapenia show more fat mass in the leg than normal older men. Future longitudinal intervention studies are needed to evaluate whether increasing muscle mass in the leg among older women and decreasing fat mass in the leg among older men influence the progress of dynapenia for older adults.

## Data Availability Statement

The raw data supporting the conclusions of this article will be made available by the authors, without undue reservation.

## Ethics Statement

The studies involving human participants were reviewed and approved by the Ethical Committee of the Graduate School of Integrated Arts and Sciences, Hiroshima University. The patients/participants provided their written informed consent to participate in this study.

## Author Contributions

HJ, ST, and RT contributed to the study design, collected, and analyzed the data. All authors contributed to the article and approved the submitted version.

## Conflict of Interest

The authors declare that the research was conducted in the absence of any commercial or financial relationships that could be construed as a potential conflict of interest.

## Publisher's Note

All claims expressed in this article are solely those of the authors and do not necessarily represent those of their affiliated organizations, or those of the publisher, the editors and the reviewers. Any product that may be evaluated in this article, or claim that may be made by its manufacturer, is not guaranteed or endorsed by the publisher.
