# Erratum: Body composition characteristics of community-dwelling older adults with dynapenia

**DOI:** 10.3389/fnut.2022.1107965

**Published:** 2022-12-20

**Authors:** Frontiers Production Office

**Affiliations:** Frontiers Media SA, Lausanne, Switzerland

**Keywords:** bioelectrical impedance analysis (BIA), body fat, leg, muscle function, muscle mass

Due to an editorial error, “Chantal Pileggi” was listed as a reviewer. The correct second reviewer is “Rumpa Boonsinsukh.”

Due to an error, the reviewer comments from reviewer “Rumpa Boonsinsukh” were not implemented in the final published article. The changes have been listed below. The authors and handling editor agree with the changes.

The article title was previously “*Body composition characteristics of community-dwelling older adults with dynapenia or sarcopenia*.” The new title is “*Body composition characteristics of community-dwelling older adults with dynapenia*.”

Schorr et al. (2018) was not cited. The reference details appear below:

“Schorr M, Dichtel LE, Gerweck AV, Valera RD, Torriani M, Miller KK, et al. Sex differences in body composition and association with cardiometabolic risk. *Biol Sex Differ*. (2018) 9:1–10. 10.1186/s13293-018-0189-3”

Schorr et al. (2018) has now been added and cited in a new sentence in **Introduction**, paragraph 2. The corrected paragraph appears below.

“Dynapenia, which is mediated by physiological neuromuscular adaptations, is influenced by increases in body fat with consequent infiltration of intramuscular fat; thus, the loss of muscle function is not merely a result of sarcopenia (14–18). A previous study evaluating skeletal muscle characteristics in dynapenia, sarcopenia, and presarcopenia demonstrated that skeletal muscle characteristics of dynapenia vary markedly from those of presarcopenia, which is defined as having low skeletal muscle index but normal muscle function (11). The abovementioned study also indicated that older adults diagnosed with dynapenia or sarcopenia have a lower knee extension torque than both normal older adults and those with presarcopenia (but not those with both dynapenia and sarcopenia) (11). Additionally, a cross-sectional study showed that older adults with dynapenia had decreased thicknesses of the rectus femoris and medial gastrocnemius muscles compared with those without dynapenia (7). Another study reported that older adults with dynapenia have a higher body mass index and fat mass than adults with sarcopenia and presarcopenia (8). The extremities may also have varying muscle and fat masses in dynapenia, sarcopenia, and presarcopenia compared with normal older adults. Moreover, it may be different in men and women, as men have a higher ratio of muscle to fat than women (19). However, appendicular muscle and fat masses of older adults with dynapenia, sarcopenia, or presarcopenia have not been compared with those of normal older adults.”

In **Methods**, Section “*Statistical analysis and sample size*,” paragraph 2, “(11 people in each group)” was missing. The corrected paragraph appears below.

“The sample size required to ensure sufficient power in the MANOVA was calculated. G^*^power 3.1.9.7 was used for this calculation (25). The parameters were set as follows: effect size = 0.15, significance level = 0.05, effect size = 0.80, number of groups = 4, and response variables = 4. The result of the calculation demonstrated that a sample of 44 people (11 people in each group) was required to correctly detect a significant difference among the groups.”

The publisher apologizes for these mistakes. The original version of this article has been updated.

